# Supplementary figures and images for: Automatic Fuzzy Logic-Based Maize Common Rust Disease Severity Predictions with Thresholding and Deep Learning (part 2 of 2)
Source: Pathogens. 2021 Jan 28;10(2):131. doi: 10.3390/pathogens10020131 (PMC7912646; doi:10.3390/pathogens10020131)

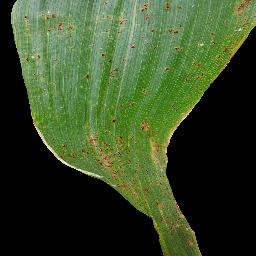

Supplement: Supplementary file 1 [file pathogens-10-00131-s001.zip › common_rust_data/train/Early_stage_common_rust/rst (116).JPG]

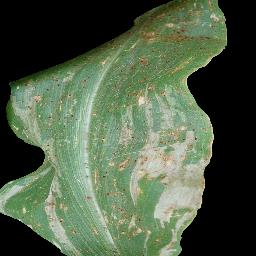

Supplement: Supplementary file 1 [file pathogens-10-00131-s001.zip › common_rust_data/train/Early_stage_common_rust/rst (117) - Copy.JPG]

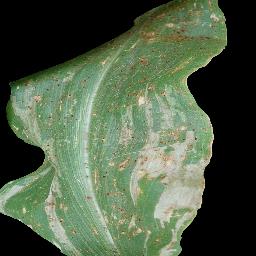

Supplement: Supplementary file 1 [file pathogens-10-00131-s001.zip › common_rust_data/train/Early_stage_common_rust/rst (117).JPG]

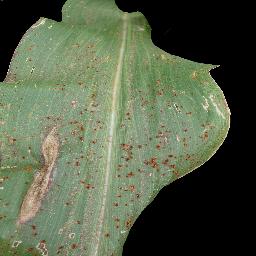

Supplement: Supplementary file 1 [file pathogens-10-00131-s001.zip › common_rust_data/train/Early_stage_common_rust/rst (118) - Copy.JPG]

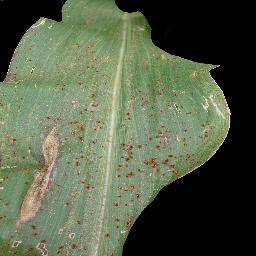

Supplement: Supplementary file 1 [file pathogens-10-00131-s001.zip › common_rust_data/train/Early_stage_common_rust/rst (118).JPG]

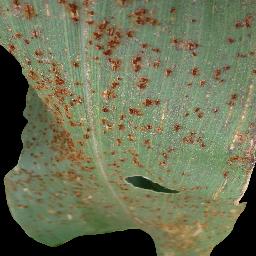

Supplement: Supplementary file 1 [file pathogens-10-00131-s001.zip › common_rust_data/train/Early_stage_common_rust/rst (119) - Copy.JPG]

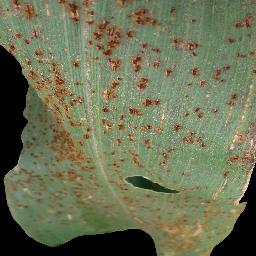

Supplement: Supplementary file 1 [file pathogens-10-00131-s001.zip › common_rust_data/train/Early_stage_common_rust/rst (119).JPG]

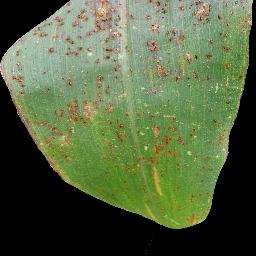

Supplement: Supplementary file 1 [file pathogens-10-00131-s001.zip › common_rust_data/train/Early_stage_common_rust/rst (12) - Copy.JPG]

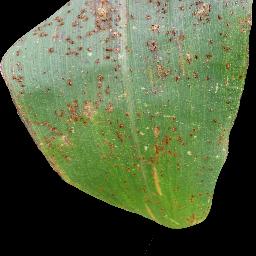

Supplement: Supplementary file 1 [file pathogens-10-00131-s001.zip › common_rust_data/train/Early_stage_common_rust/rst (12).JPG]

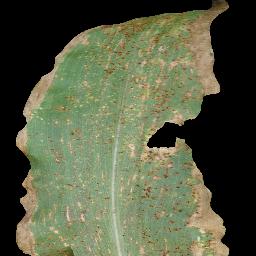

Supplement: Supplementary file 1 [file pathogens-10-00131-s001.zip › common_rust_data/train/Early_stage_common_rust/rst (120) - Copy.JPG]

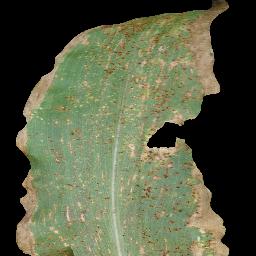

Supplement: Supplementary file 1 [file pathogens-10-00131-s001.zip › common_rust_data/train/Early_stage_common_rust/rst (120).JPG]

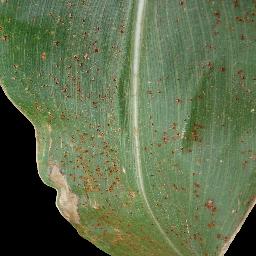

Supplement: Supplementary file 1 [file pathogens-10-00131-s001.zip › common_rust_data/train/Early_stage_common_rust/rst (121) - Copy.JPG]

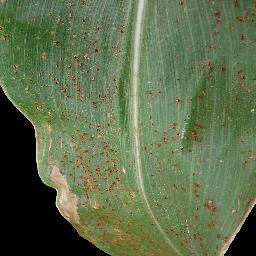

Supplement: Supplementary file 1 [file pathogens-10-00131-s001.zip › common_rust_data/train/Early_stage_common_rust/rst (121).JPG]

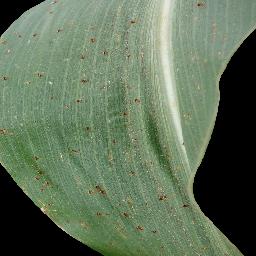

Supplement: Supplementary file 1 [file pathogens-10-00131-s001.zip › common_rust_data/train/Early_stage_common_rust/rst (122) - Copy.JPG]

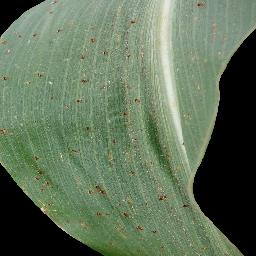

Supplement: Supplementary file 1 [file pathogens-10-00131-s001.zip › common_rust_data/train/Early_stage_common_rust/rst (122).JPG]

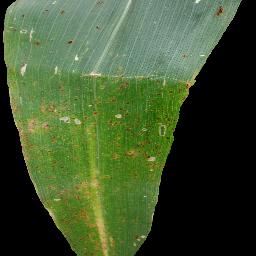

Supplement: Supplementary file 1 [file pathogens-10-00131-s001.zip › common_rust_data/train/Early_stage_common_rust/rst (123) - Copy.JPG]

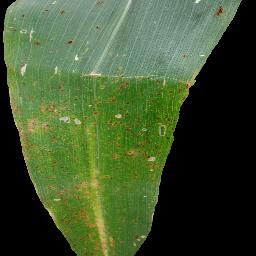

Supplement: Supplementary file 1 [file pathogens-10-00131-s001.zip › common_rust_data/train/Early_stage_common_rust/rst (123).JPG]

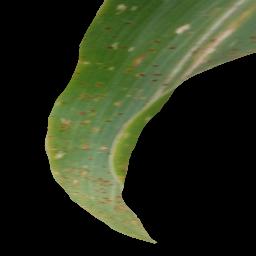

Supplement: Supplementary file 1 [file pathogens-10-00131-s001.zip › common_rust_data/train/Early_stage_common_rust/rst (124) - Copy.JPG]

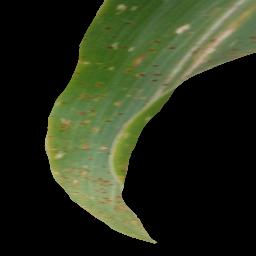

Supplement: Supplementary file 1 [file pathogens-10-00131-s001.zip › common_rust_data/train/Early_stage_common_rust/rst (124).JPG]

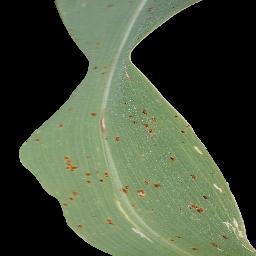

Supplement: Supplementary file 1 [file pathogens-10-00131-s001.zip › common_rust_data/train/Early_stage_common_rust/rst (125) - Copy.JPG]

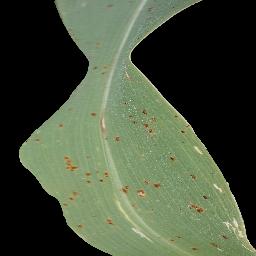

Supplement: Supplementary file 1 [file pathogens-10-00131-s001.zip › common_rust_data/train/Early_stage_common_rust/rst (125).JPG]

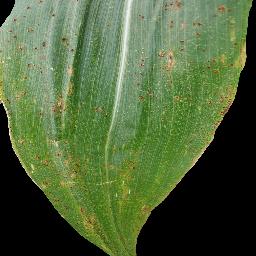

Supplement: Supplementary file 1 [file pathogens-10-00131-s001.zip › common_rust_data/train/Early_stage_common_rust/rst (126) - Copy.JPG]

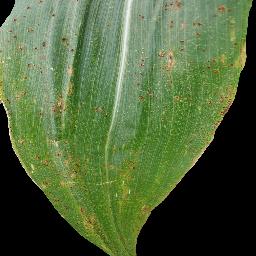

Supplement: Supplementary file 1 [file pathogens-10-00131-s001.zip › common_rust_data/train/Early_stage_common_rust/rst (126).JPG]

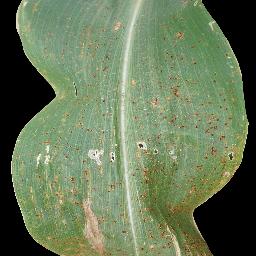

Supplement: Supplementary file 1 [file pathogens-10-00131-s001.zip › common_rust_data/train/Early_stage_common_rust/rst (127) - Copy.JPG]

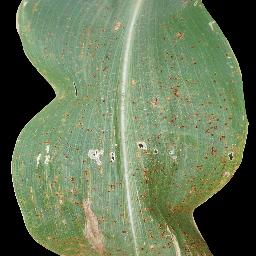

Supplement: Supplementary file 1 [file pathogens-10-00131-s001.zip › common_rust_data/train/Early_stage_common_rust/rst (127).JPG]

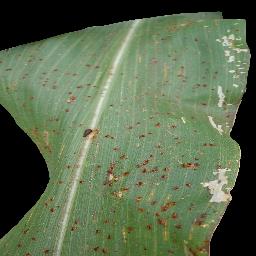

Supplement: Supplementary file 1 [file pathogens-10-00131-s001.zip › common_rust_data/train/Early_stage_common_rust/rst (128) - Copy.JPG]

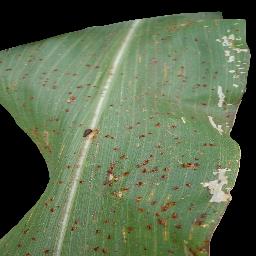

Supplement: Supplementary file 1 [file pathogens-10-00131-s001.zip › common_rust_data/train/Early_stage_common_rust/rst (128).JPG]

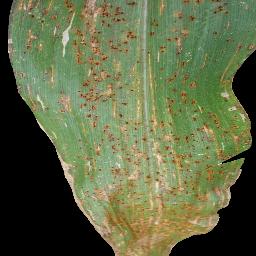

Supplement: Supplementary file 1 [file pathogens-10-00131-s001.zip › common_rust_data/train/Early_stage_common_rust/rst (129) - Copy.JPG]

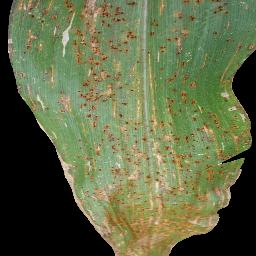

Supplement: Supplementary file 1 [file pathogens-10-00131-s001.zip › common_rust_data/train/Early_stage_common_rust/rst (129).JPG]

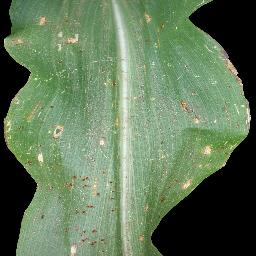

Supplement: Supplementary file 1 [file pathogens-10-00131-s001.zip › common_rust_data/train/Early_stage_common_rust/rst (13) - Copy.JPG]

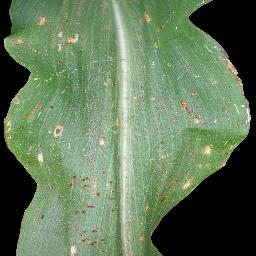

Supplement: Supplementary file 1 [file pathogens-10-00131-s001.zip › common_rust_data/train/Early_stage_common_rust/rst (13).JPG]

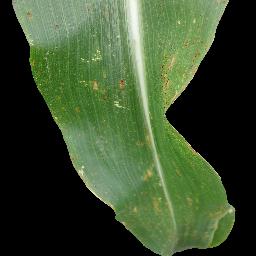

Supplement: Supplementary file 1 [file pathogens-10-00131-s001.zip › common_rust_data/train/Early_stage_common_rust/rst (130) - Copy.JPG]

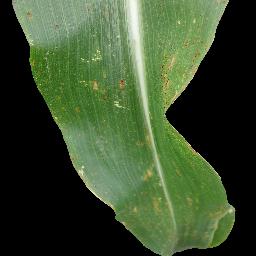

Supplement: Supplementary file 1 [file pathogens-10-00131-s001.zip › common_rust_data/train/Early_stage_common_rust/rst (130).JPG]

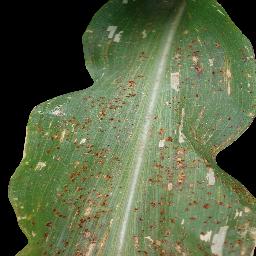

Supplement: Supplementary file 1 [file pathogens-10-00131-s001.zip › common_rust_data/train/Early_stage_common_rust/rst (131) - Copy.JPG]

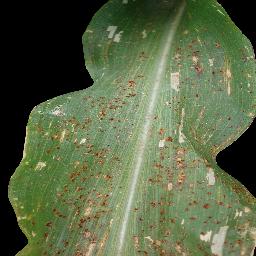

Supplement: Supplementary file 1 [file pathogens-10-00131-s001.zip › common_rust_data/train/Early_stage_common_rust/rst (131).JPG]

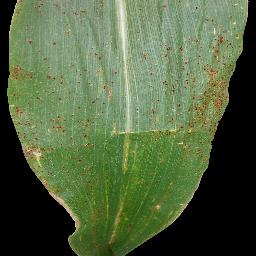

Supplement: Supplementary file 1 [file pathogens-10-00131-s001.zip › common_rust_data/train/Early_stage_common_rust/rst (132) - Copy.JPG]

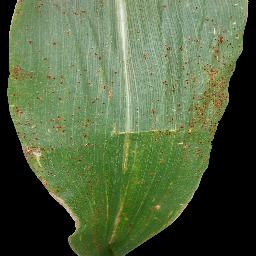

Supplement: Supplementary file 1 [file pathogens-10-00131-s001.zip › common_rust_data/train/Early_stage_common_rust/rst (132).JPG]

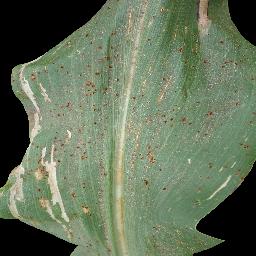

Supplement: Supplementary file 1 [file pathogens-10-00131-s001.zip › common_rust_data/train/Early_stage_common_rust/rst (133) - Copy.JPG]

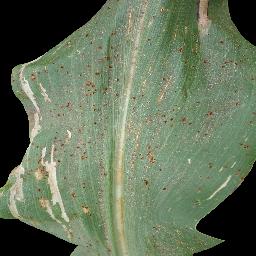

Supplement: Supplementary file 1 [file pathogens-10-00131-s001.zip › common_rust_data/train/Early_stage_common_rust/rst (133).JPG]

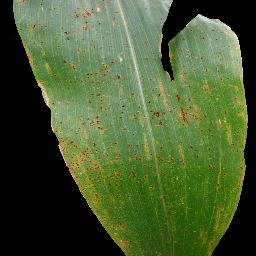

Supplement: Supplementary file 1 [file pathogens-10-00131-s001.zip › common_rust_data/train/Early_stage_common_rust/rst (134) - Copy.JPG]

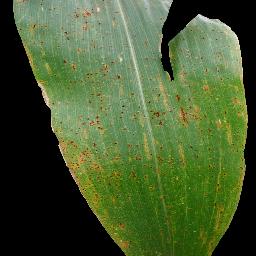

Supplement: Supplementary file 1 [file pathogens-10-00131-s001.zip › common_rust_data/train/Early_stage_common_rust/rst (134).JPG]

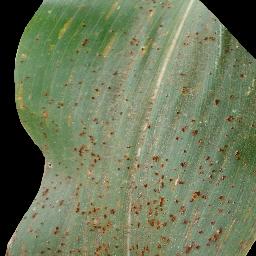

Supplement: Supplementary file 1 [file pathogens-10-00131-s001.zip › common_rust_data/train/Early_stage_common_rust/rst (135) - Copy.JPG]

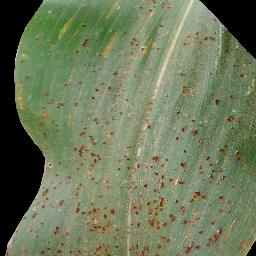

Supplement: Supplementary file 1 [file pathogens-10-00131-s001.zip › common_rust_data/train/Early_stage_common_rust/rst (135).JPG]

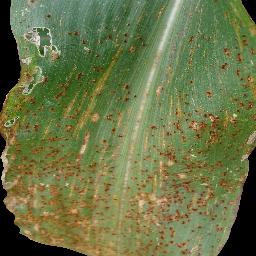

Supplement: Supplementary file 1 [file pathogens-10-00131-s001.zip › common_rust_data/train/Early_stage_common_rust/rst (136) - Copy.JPG]

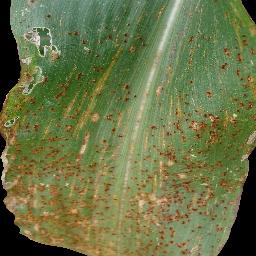

Supplement: Supplementary file 1 [file pathogens-10-00131-s001.zip › common_rust_data/train/Early_stage_common_rust/rst (136).JPG]

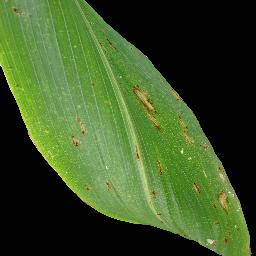

Supplement: Supplementary file 1 [file pathogens-10-00131-s001.zip › common_rust_data/train/Early_stage_common_rust/rst (137) - Copy.JPG]

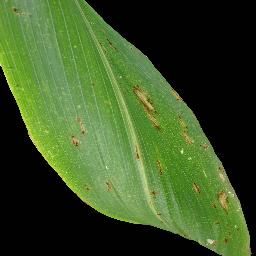

Supplement: Supplementary file 1 [file pathogens-10-00131-s001.zip › common_rust_data/train/Early_stage_common_rust/rst (137).JPG]

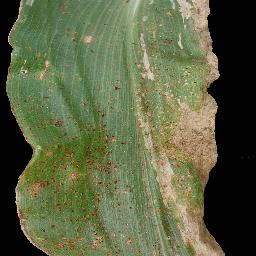

Supplement: Supplementary file 1 [file pathogens-10-00131-s001.zip › common_rust_data/train/Early_stage_common_rust/rst (138) - Copy.JPG]

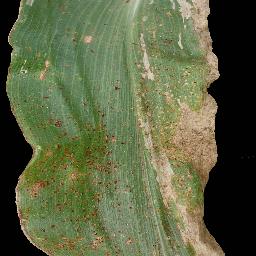

Supplement: Supplementary file 1 [file pathogens-10-00131-s001.zip › common_rust_data/train/Early_stage_common_rust/rst (138).JPG]

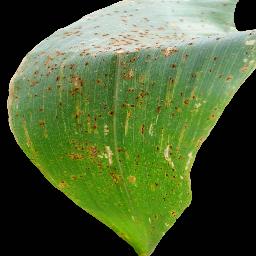

Supplement: Supplementary file 1 [file pathogens-10-00131-s001.zip › common_rust_data/train/Early_stage_common_rust/rst (139) - Copy.JPG]

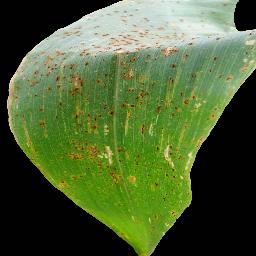

Supplement: Supplementary file 1 [file pathogens-10-00131-s001.zip › common_rust_data/train/Early_stage_common_rust/rst (139).JPG]

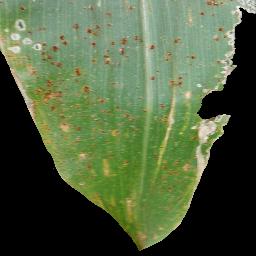

Supplement: Supplementary file 1 [file pathogens-10-00131-s001.zip › common_rust_data/train/Early_stage_common_rust/rst (14) - Copy.JPG]

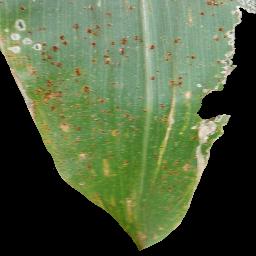

Supplement: Supplementary file 1 [file pathogens-10-00131-s001.zip › common_rust_data/train/Early_stage_common_rust/rst (14).JPG]

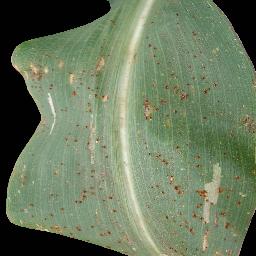

Supplement: Supplementary file 1 [file pathogens-10-00131-s001.zip › common_rust_data/train/Early_stage_common_rust/rst (140) - Copy.JPG]

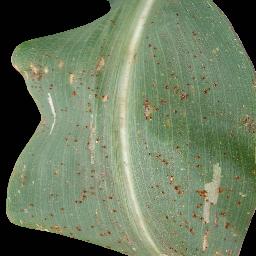

Supplement: Supplementary file 1 [file pathogens-10-00131-s001.zip › common_rust_data/train/Early_stage_common_rust/rst (140).JPG]

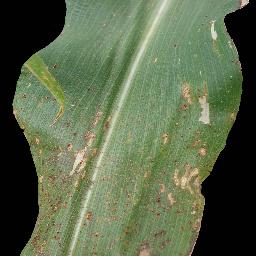

Supplement: Supplementary file 1 [file pathogens-10-00131-s001.zip › common_rust_data/train/Early_stage_common_rust/rst (141) - Copy.JPG]

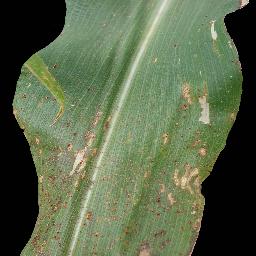

Supplement: Supplementary file 1 [file pathogens-10-00131-s001.zip › common_rust_data/train/Early_stage_common_rust/rst (141).JPG]

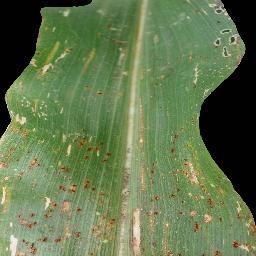

Supplement: Supplementary file 1 [file pathogens-10-00131-s001.zip › common_rust_data/train/Early_stage_common_rust/rst (142) - Copy.JPG]

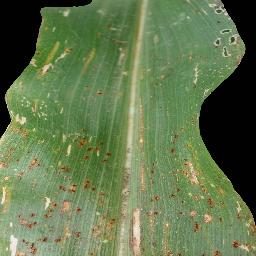

Supplement: Supplementary file 1 [file pathogens-10-00131-s001.zip › common_rust_data/train/Early_stage_common_rust/rst (142).JPG]

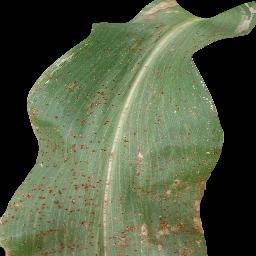

Supplement: Supplementary file 1 [file pathogens-10-00131-s001.zip › common_rust_data/train/Early_stage_common_rust/rst (143) - Copy.JPG]

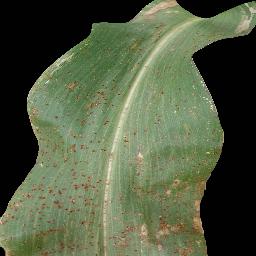

Supplement: Supplementary file 1 [file pathogens-10-00131-s001.zip › common_rust_data/train/Early_stage_common_rust/rst (143).JPG]

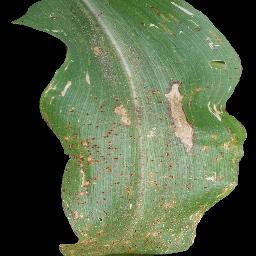

Supplement: Supplementary file 1 [file pathogens-10-00131-s001.zip › common_rust_data/train/Early_stage_common_rust/rst (144) - Copy.JPG]

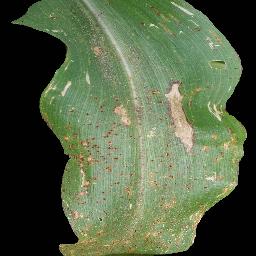

Supplement: Supplementary file 1 [file pathogens-10-00131-s001.zip › common_rust_data/train/Early_stage_common_rust/rst (144).JPG]

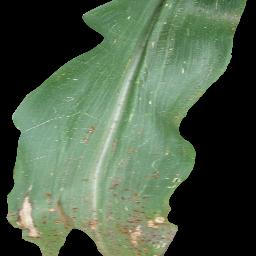

Supplement: Supplementary file 1 [file pathogens-10-00131-s001.zip › common_rust_data/train/Early_stage_common_rust/rst (145) - Copy.JPG]

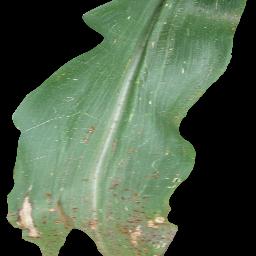

Supplement: Supplementary file 1 [file pathogens-10-00131-s001.zip › common_rust_data/train/Early_stage_common_rust/rst (145).JPG]

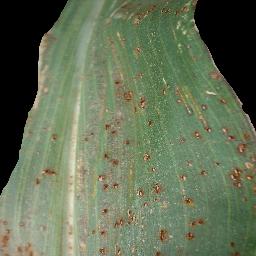

Supplement: Supplementary file 1 [file pathogens-10-00131-s001.zip › common_rust_data/train/Early_stage_common_rust/rst (146) - Copy.JPG]

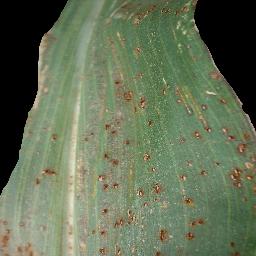

Supplement: Supplementary file 1 [file pathogens-10-00131-s001.zip › common_rust_data/train/Early_stage_common_rust/rst (146).JPG]

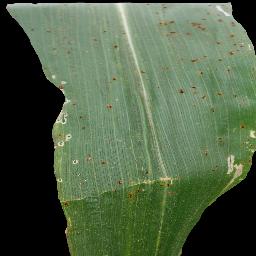

Supplement: Supplementary file 1 [file pathogens-10-00131-s001.zip › common_rust_data/train/Early_stage_common_rust/rst (147) - Copy.JPG]

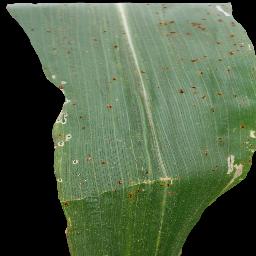

Supplement: Supplementary file 1 [file pathogens-10-00131-s001.zip › common_rust_data/train/Early_stage_common_rust/rst (147).JPG]

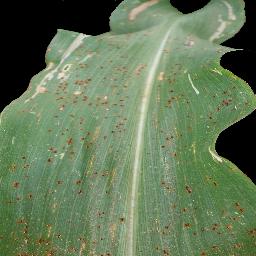

Supplement: Supplementary file 1 [file pathogens-10-00131-s001.zip › common_rust_data/train/Early_stage_common_rust/rst (148) - Copy.JPG]

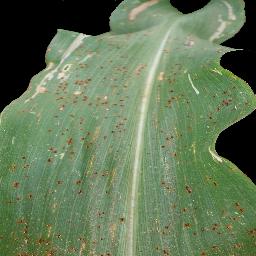

Supplement: Supplementary file 1 [file pathogens-10-00131-s001.zip › common_rust_data/train/Early_stage_common_rust/rst (148).JPG]

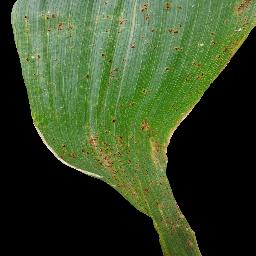

Supplement: Supplementary file 1 [file pathogens-10-00131-s001.zip › common_rust_data/train/Early_stage_common_rust/rst (149) - Copy.JPG]

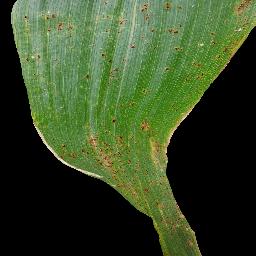

Supplement: Supplementary file 1 [file pathogens-10-00131-s001.zip › common_rust_data/train/Early_stage_common_rust/rst (149).JPG]

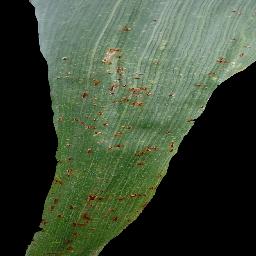

Supplement: Supplementary file 1 [file pathogens-10-00131-s001.zip › common_rust_data/train/Early_stage_common_rust/rst (15) - Copy.JPG]

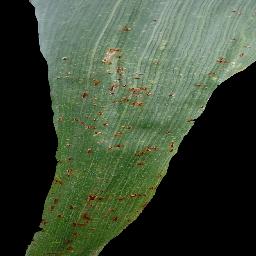

Supplement: Supplementary file 1 [file pathogens-10-00131-s001.zip › common_rust_data/train/Early_stage_common_rust/rst (15).JPG]

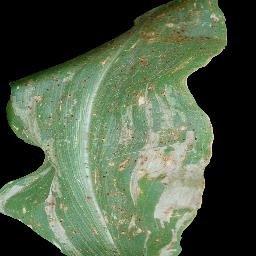

Supplement: Supplementary file 1 [file pathogens-10-00131-s001.zip › common_rust_data/train/Early_stage_common_rust/rst (150) - Copy.JPG]

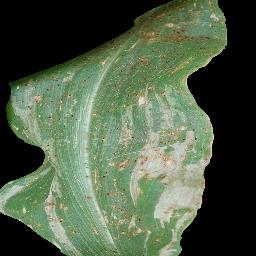

Supplement: Supplementary file 1 [file pathogens-10-00131-s001.zip › common_rust_data/train/Early_stage_common_rust/rst (150).JPG]

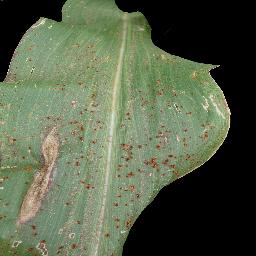

Supplement: Supplementary file 1 [file pathogens-10-00131-s001.zip › common_rust_data/train/Early_stage_common_rust/rst (151) - Copy.JPG]

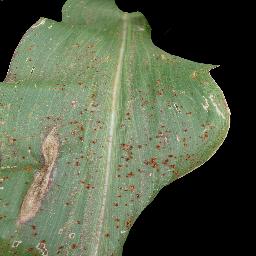

Supplement: Supplementary file 1 [file pathogens-10-00131-s001.zip › common_rust_data/train/Early_stage_common_rust/rst (151).JPG]

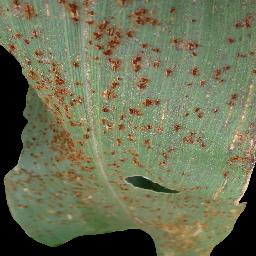

Supplement: Supplementary file 1 [file pathogens-10-00131-s001.zip › common_rust_data/train/Early_stage_common_rust/rst (152) - Copy.JPG]

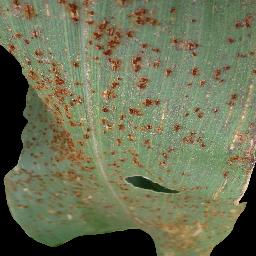

Supplement: Supplementary file 1 [file pathogens-10-00131-s001.zip › common_rust_data/train/Early_stage_common_rust/rst (152).JPG]

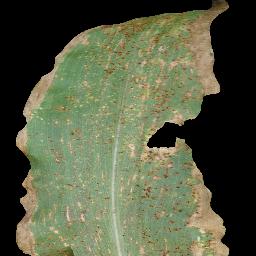

Supplement: Supplementary file 1 [file pathogens-10-00131-s001.zip › common_rust_data/train/Early_stage_common_rust/rst (153) - Copy.JPG]

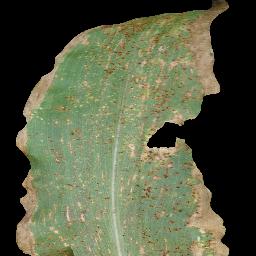

Supplement: Supplementary file 1 [file pathogens-10-00131-s001.zip › common_rust_data/train/Early_stage_common_rust/rst (153).JPG]

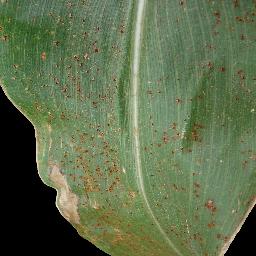

Supplement: Supplementary file 1 [file pathogens-10-00131-s001.zip › common_rust_data/train/Early_stage_common_rust/rst (154) - Copy.JPG]

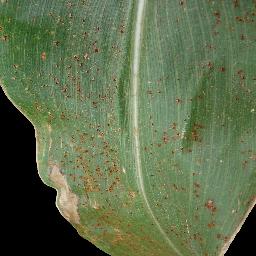

Supplement: Supplementary file 1 [file pathogens-10-00131-s001.zip › common_rust_data/train/Early_stage_common_rust/rst (154).JPG]

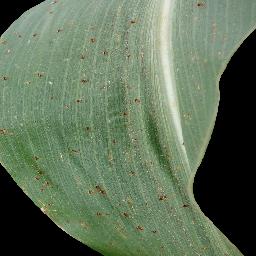

Supplement: Supplementary file 1 [file pathogens-10-00131-s001.zip › common_rust_data/train/Early_stage_common_rust/rst (155) - Copy.JPG]
