# Supplementary figures and images for: Automatic Fuzzy Logic-Based Maize Common Rust Disease Severity Predictions with Thresholding and Deep Learning (part 1 of 2)
Source: Pathogens. 2021 Jan 28;10(2):131. doi: 10.3390/pathogens10020131 (PMC7912646; doi:10.3390/pathogens10020131)

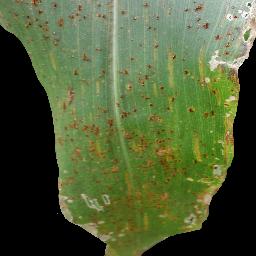

Supplement: Supplementary file 1 [file pathogens-10-00131-s001.zip › common_rust_data/test/Early_stage_common_rust/rst (36) - Copy.JPG]

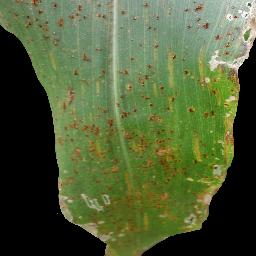

Supplement: Supplementary file 1 [file pathogens-10-00131-s001.zip › common_rust_data/test/Early_stage_common_rust/rst (36).JPG]

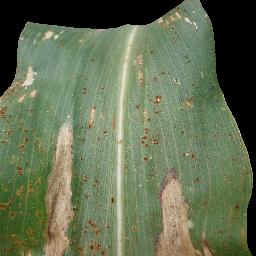

Supplement: Supplementary file 1 [file pathogens-10-00131-s001.zip › common_rust_data/test/Early_stage_common_rust/rst (37) - Copy.JPG]

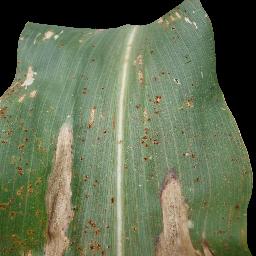

Supplement: Supplementary file 1 [file pathogens-10-00131-s001.zip › common_rust_data/test/Early_stage_common_rust/rst (37).JPG]

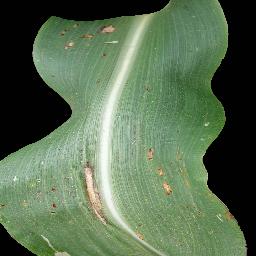

Supplement: Supplementary file 1 [file pathogens-10-00131-s001.zip › common_rust_data/test/Early_stage_common_rust/rst (38) - Copy.JPG]

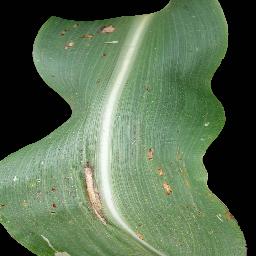

Supplement: Supplementary file 1 [file pathogens-10-00131-s001.zip › common_rust_data/test/Early_stage_common_rust/rst (38).JPG]

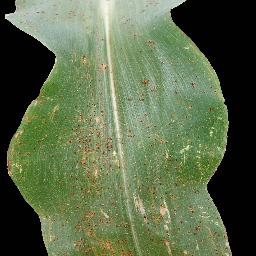

Supplement: Supplementary file 1 [file pathogens-10-00131-s001.zip › common_rust_data/test/Early_stage_common_rust/rst (39) - Copy.JPG]

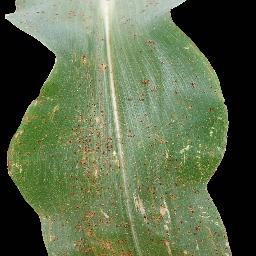

Supplement: Supplementary file 1 [file pathogens-10-00131-s001.zip › common_rust_data/test/Early_stage_common_rust/rst (39).JPG]

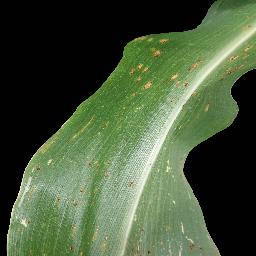

Supplement: Supplementary file 1 [file pathogens-10-00131-s001.zip › common_rust_data/test/Early_stage_common_rust/rst (40) - Copy.JPG]

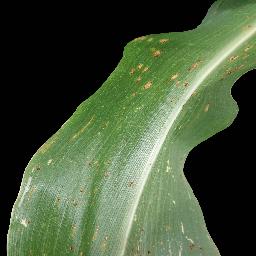

Supplement: Supplementary file 1 [file pathogens-10-00131-s001.zip › common_rust_data/test/Early_stage_common_rust/rst (40).JPG]

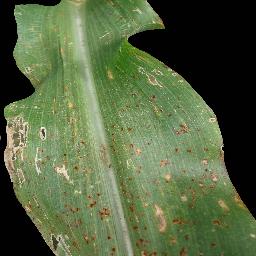

Supplement: Supplementary file 1 [file pathogens-10-00131-s001.zip › common_rust_data/test/Early_stage_common_rust/rst (41) - Copy.JPG]

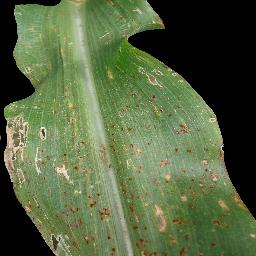

Supplement: Supplementary file 1 [file pathogens-10-00131-s001.zip › common_rust_data/test/Early_stage_common_rust/rst (41).JPG]

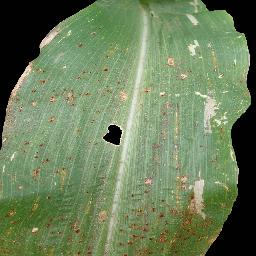

Supplement: Supplementary file 1 [file pathogens-10-00131-s001.zip › common_rust_data/test/Early_stage_common_rust/rst (42) - Copy.JPG]

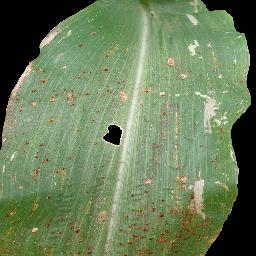

Supplement: Supplementary file 1 [file pathogens-10-00131-s001.zip › common_rust_data/test/Early_stage_common_rust/rst (42).JPG]

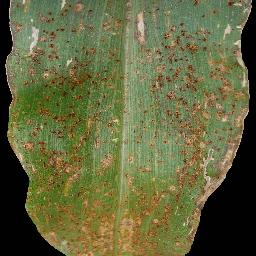

Supplement: Supplementary file 1 [file pathogens-10-00131-s001.zip › common_rust_data/test/Early_stage_common_rust/rst (43) - Copy.JPG]

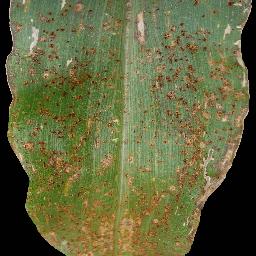

Supplement: Supplementary file 1 [file pathogens-10-00131-s001.zip › common_rust_data/test/Early_stage_common_rust/rst (43).JPG]

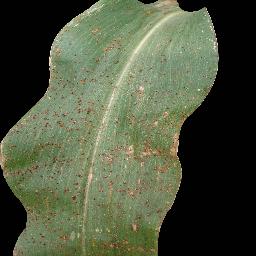

Supplement: Supplementary file 1 [file pathogens-10-00131-s001.zip › common_rust_data/test/Early_stage_common_rust/rst (44) - Copy.JPG]

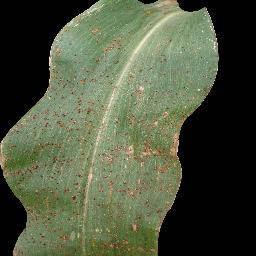

Supplement: Supplementary file 1 [file pathogens-10-00131-s001.zip › common_rust_data/test/Early_stage_common_rust/rst (44).JPG]

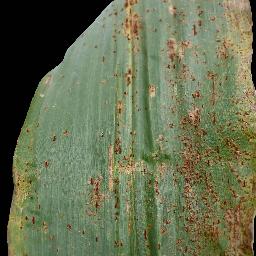

Supplement: Supplementary file 1 [file pathogens-10-00131-s001.zip › common_rust_data/test/Early_stage_common_rust/rst (45) - Copy.JPG]

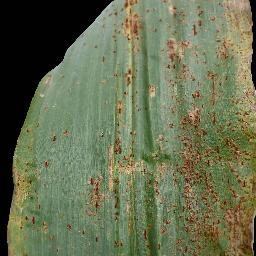

Supplement: Supplementary file 1 [file pathogens-10-00131-s001.zip › common_rust_data/test/Early_stage_common_rust/rst (45).JPG]

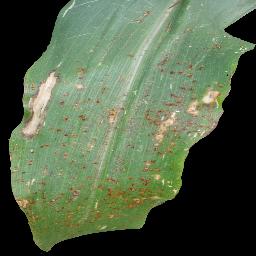

Supplement: Supplementary file 1 [file pathogens-10-00131-s001.zip › common_rust_data/test/Early_stage_common_rust/rst (46) - Copy.JPG]

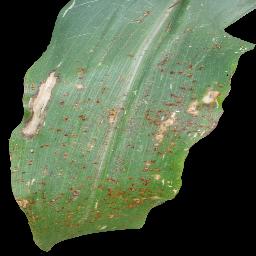

Supplement: Supplementary file 1 [file pathogens-10-00131-s001.zip › common_rust_data/test/Early_stage_common_rust/rst (46).JPG]

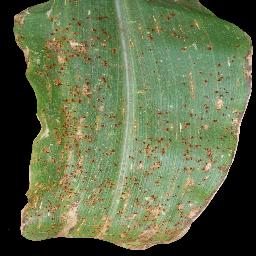

Supplement: Supplementary file 1 [file pathogens-10-00131-s001.zip › common_rust_data/test/Early_stage_common_rust/rst (47) - Copy.JPG]

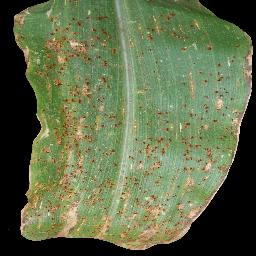

Supplement: Supplementary file 1 [file pathogens-10-00131-s001.zip › common_rust_data/test/Early_stage_common_rust/rst (47).JPG]

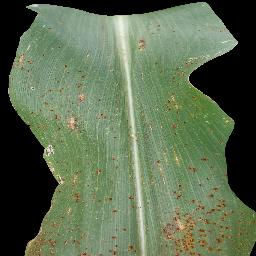

Supplement: Supplementary file 1 [file pathogens-10-00131-s001.zip › common_rust_data/test/Early_stage_common_rust/rst (48) - Copy.JPG]

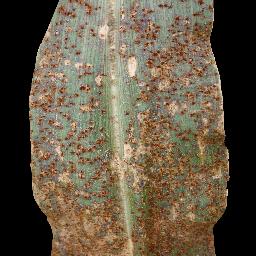

Supplement: Supplementary file 1 [file pathogens-10-00131-s001.zip › common_rust_data/test/Late_stage_common_rust/RS_Rust 2470.JPG]

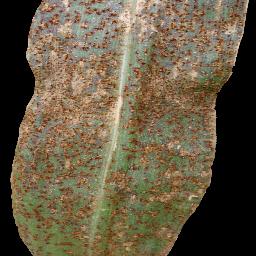

Supplement: Supplementary file 1 [file pathogens-10-00131-s001.zip › common_rust_data/test/Late_stage_common_rust/RS_Rust 2471.JPG]

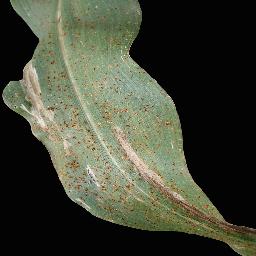

Supplement: Supplementary file 1 [file pathogens-10-00131-s001.zip › common_rust_data/test/Late_stage_common_rust/rust (1).JPG]

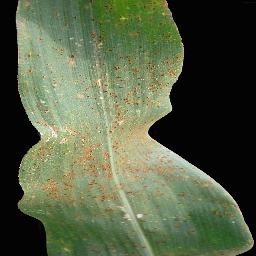

Supplement: Supplementary file 1 [file pathogens-10-00131-s001.zip › common_rust_data/test/Late_stage_common_rust/rust (10).JPG]

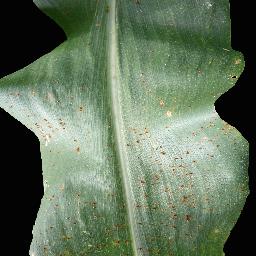

Supplement: Supplementary file 1 [file pathogens-10-00131-s001.zip › common_rust_data/test/Late_stage_common_rust/rust (11).JPG]

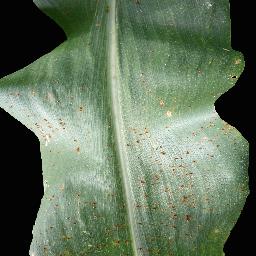

Supplement: Supplementary file 1 [file pathogens-10-00131-s001.zip › common_rust_data/test/Late_stage_common_rust/rust (12).JPG]

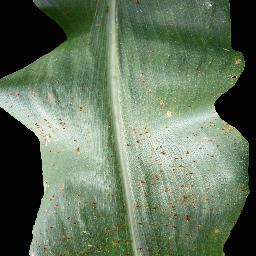

Supplement: Supplementary file 1 [file pathogens-10-00131-s001.zip › common_rust_data/test/Late_stage_common_rust/rust (13).JPG]

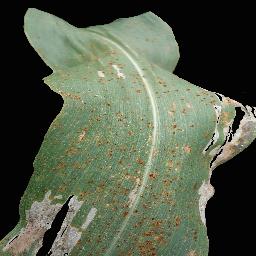

Supplement: Supplementary file 1 [file pathogens-10-00131-s001.zip › common_rust_data/test/Late_stage_common_rust/rust (15).JPG]

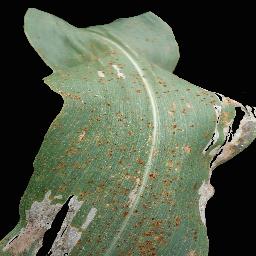

Supplement: Supplementary file 1 [file pathogens-10-00131-s001.zip › common_rust_data/test/Late_stage_common_rust/rust (16).JPG]

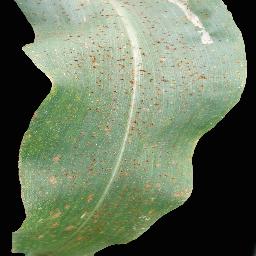

Supplement: Supplementary file 1 [file pathogens-10-00131-s001.zip › common_rust_data/test/Late_stage_common_rust/rust (19).JPG]

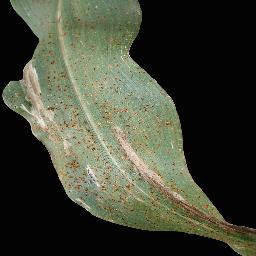

Supplement: Supplementary file 1 [file pathogens-10-00131-s001.zip › common_rust_data/test/Late_stage_common_rust/rust (2).JPG]

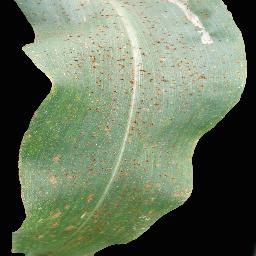

Supplement: Supplementary file 1 [file pathogens-10-00131-s001.zip › common_rust_data/test/Late_stage_common_rust/rust (20).JPG]

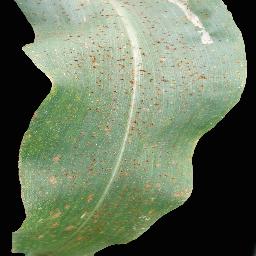

Supplement: Supplementary file 1 [file pathogens-10-00131-s001.zip › common_rust_data/test/Late_stage_common_rust/rust (21).JPG]

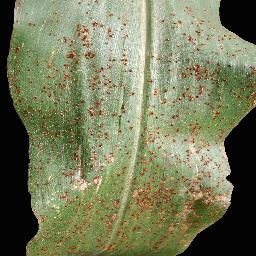

Supplement: Supplementary file 1 [file pathogens-10-00131-s001.zip › common_rust_data/test/Late_stage_common_rust/rust (27).JPG]

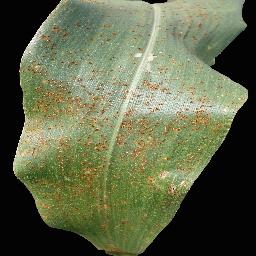

Supplement: Supplementary file 1 [file pathogens-10-00131-s001.zip › common_rust_data/test/Late_stage_common_rust/rust (3).JPG]

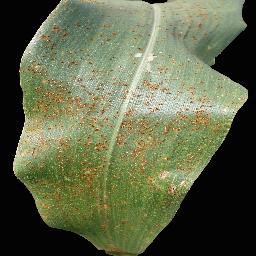

Supplement: Supplementary file 1 [file pathogens-10-00131-s001.zip › common_rust_data/test/Late_stage_common_rust/rust (4).JPG]

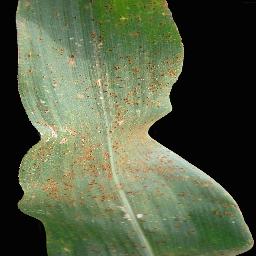

Supplement: Supplementary file 1 [file pathogens-10-00131-s001.zip › common_rust_data/test/Late_stage_common_rust/rust (8).JPG]

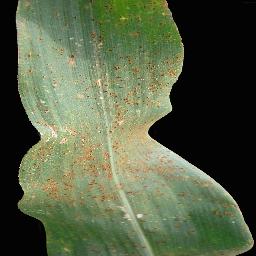

Supplement: Supplementary file 1 [file pathogens-10-00131-s001.zip › common_rust_data/test/Late_stage_common_rust/rust (9).JPG]

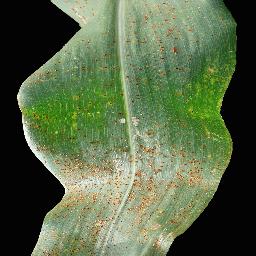

Supplement: Supplementary file 1 [file pathogens-10-00131-s001.zip › common_rust_data/test/Middle_stage_common_rust/rst (1).JPG]

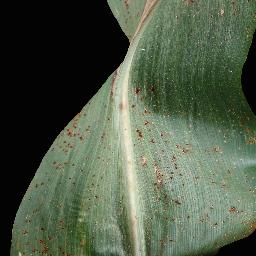

Supplement: Supplementary file 1 [file pathogens-10-00131-s001.zip › common_rust_data/test/Middle_stage_common_rust/rst (10).JPG]

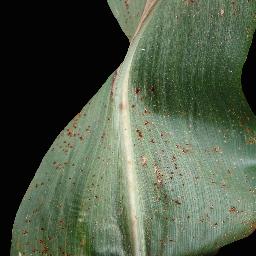

Supplement: Supplementary file 1 [file pathogens-10-00131-s001.zip › common_rust_data/test/Middle_stage_common_rust/rst (11).JPG]

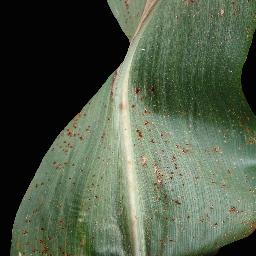

Supplement: Supplementary file 1 [file pathogens-10-00131-s001.zip › common_rust_data/test/Middle_stage_common_rust/rst (12).JPG]

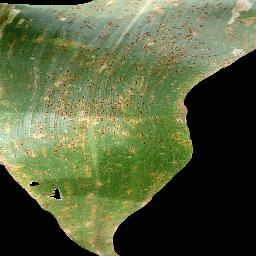

Supplement: Supplementary file 1 [file pathogens-10-00131-s001.zip › common_rust_data/test/Middle_stage_common_rust/rst (13).JPG]

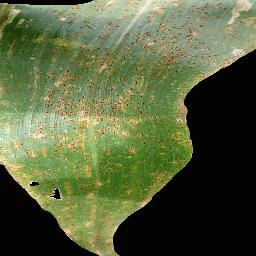

Supplement: Supplementary file 1 [file pathogens-10-00131-s001.zip › common_rust_data/test/Middle_stage_common_rust/rst (15).JPG]

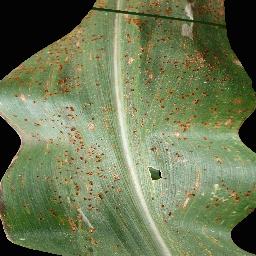

Supplement: Supplementary file 1 [file pathogens-10-00131-s001.zip › common_rust_data/test/Middle_stage_common_rust/rst (17).JPG]

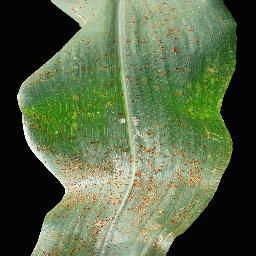

Supplement: Supplementary file 1 [file pathogens-10-00131-s001.zip › common_rust_data/test/Middle_stage_common_rust/rst (2).JPG]

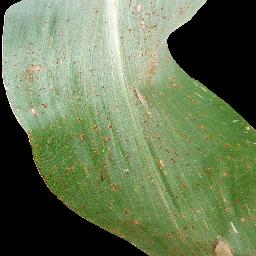

Supplement: Supplementary file 1 [file pathogens-10-00131-s001.zip › common_rust_data/test/Middle_stage_common_rust/rst (21).JPG]

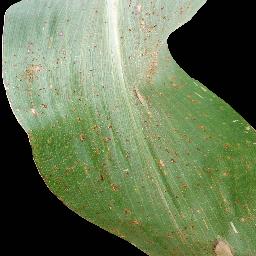

Supplement: Supplementary file 1 [file pathogens-10-00131-s001.zip › common_rust_data/test/Middle_stage_common_rust/rst (22).JPG]

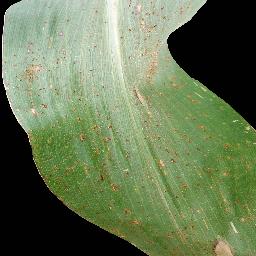

Supplement: Supplementary file 1 [file pathogens-10-00131-s001.zip › common_rust_data/test/Middle_stage_common_rust/rst (23).JPG]

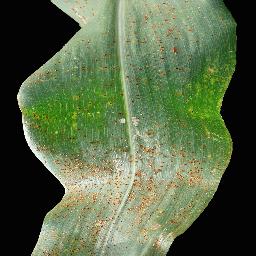

Supplement: Supplementary file 1 [file pathogens-10-00131-s001.zip › common_rust_data/test/Middle_stage_common_rust/rst (3).JPG]

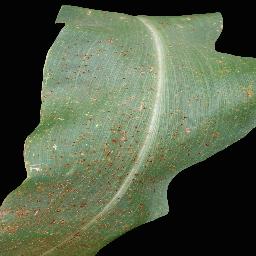

Supplement: Supplementary file 1 [file pathogens-10-00131-s001.zip › common_rust_data/test/Middle_stage_common_rust/rst (5).JPG]

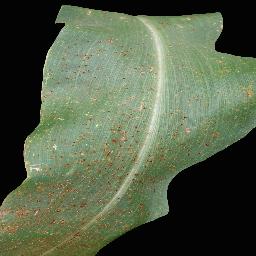

Supplement: Supplementary file 1 [file pathogens-10-00131-s001.zip › common_rust_data/test/Middle_stage_common_rust/rst (7).JPG]

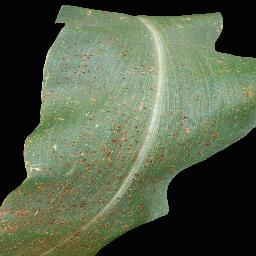

Supplement: Supplementary file 1 [file pathogens-10-00131-s001.zip › common_rust_data/test/Middle_stage_common_rust/rst (8).JPG]

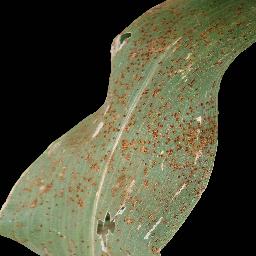

Supplement: Supplementary file 1 [file pathogens-10-00131-s001.zip › common_rust_data/test/Middle_stage_common_rust/rust (180).JPG]

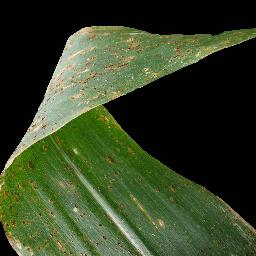

Supplement: Supplementary file 1 [file pathogens-10-00131-s001.zip › common_rust_data/test/Middle_stage_common_rust/rust (184).JPG]

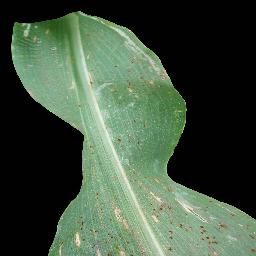

Supplement: Supplementary file 1 [file pathogens-10-00131-s001.zip › common_rust_data/test/Middle_stage_common_rust/rust (231).JPG]

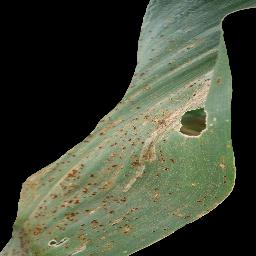

Supplement: Supplementary file 1 [file pathogens-10-00131-s001.zip › common_rust_data/train/Early_stage_common_rust/rst (1) - Copy.JPG]

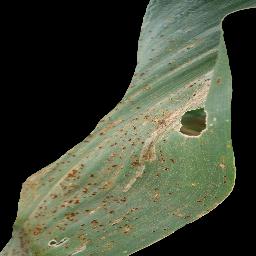

Supplement: Supplementary file 1 [file pathogens-10-00131-s001.zip › common_rust_data/train/Early_stage_common_rust/rst (1).JPG]

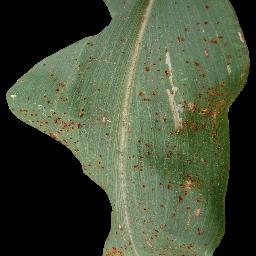

Supplement: Supplementary file 1 [file pathogens-10-00131-s001.zip › common_rust_data/train/Early_stage_common_rust/rst (10) - Copy.JPG]

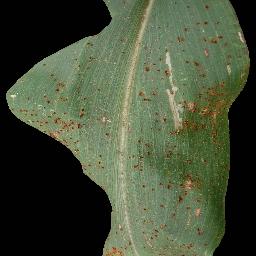

Supplement: Supplementary file 1 [file pathogens-10-00131-s001.zip › common_rust_data/train/Early_stage_common_rust/rst (10).JPG]

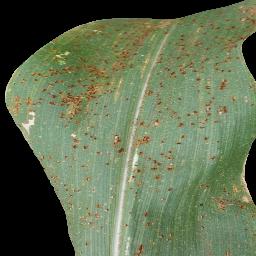

Supplement: Supplementary file 1 [file pathogens-10-00131-s001.zip › common_rust_data/train/Early_stage_common_rust/rst (100) - Copy.JPG]

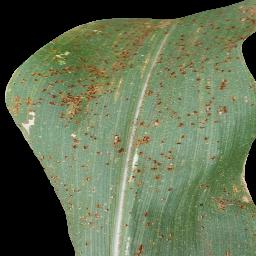

Supplement: Supplementary file 1 [file pathogens-10-00131-s001.zip › common_rust_data/train/Early_stage_common_rust/rst (100).JPG]

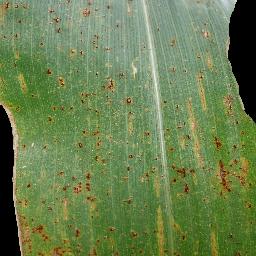

Supplement: Supplementary file 1 [file pathogens-10-00131-s001.zip › common_rust_data/train/Early_stage_common_rust/rst (101) - Copy.JPG]

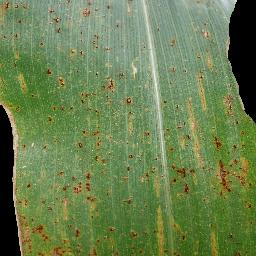

Supplement: Supplementary file 1 [file pathogens-10-00131-s001.zip › common_rust_data/train/Early_stage_common_rust/rst (101).JPG]

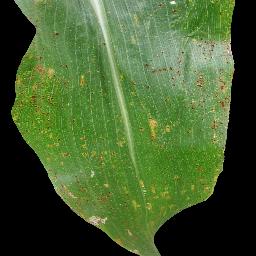

Supplement: Supplementary file 1 [file pathogens-10-00131-s001.zip › common_rust_data/train/Early_stage_common_rust/rst (102) - Copy.JPG]

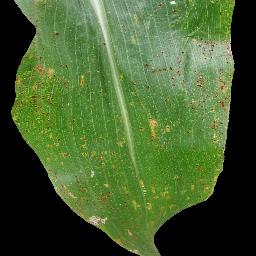

Supplement: Supplementary file 1 [file pathogens-10-00131-s001.zip › common_rust_data/train/Early_stage_common_rust/rst (102).JPG]

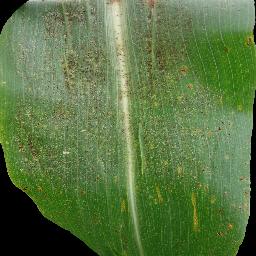

Supplement: Supplementary file 1 [file pathogens-10-00131-s001.zip › common_rust_data/train/Early_stage_common_rust/rst (103) - Copy.JPG]

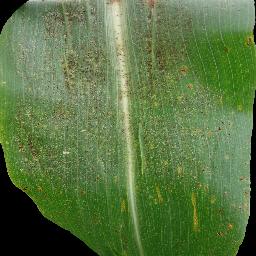

Supplement: Supplementary file 1 [file pathogens-10-00131-s001.zip › common_rust_data/train/Early_stage_common_rust/rst (103).JPG]

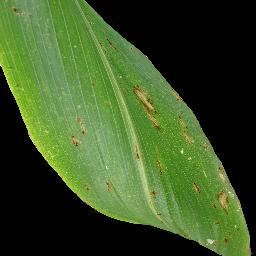

Supplement: Supplementary file 1 [file pathogens-10-00131-s001.zip › common_rust_data/train/Early_stage_common_rust/rst (104) - Copy.JPG]

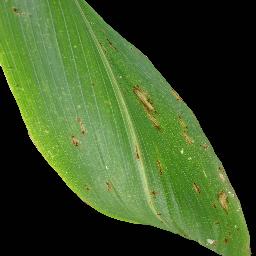

Supplement: Supplementary file 1 [file pathogens-10-00131-s001.zip › common_rust_data/train/Early_stage_common_rust/rst (104).JPG]

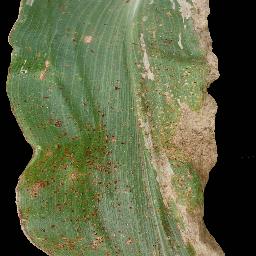

Supplement: Supplementary file 1 [file pathogens-10-00131-s001.zip › common_rust_data/train/Early_stage_common_rust/rst (105) - Copy.JPG]

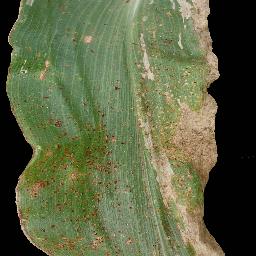

Supplement: Supplementary file 1 [file pathogens-10-00131-s001.zip › common_rust_data/train/Early_stage_common_rust/rst (105).JPG]

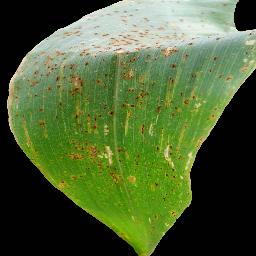

Supplement: Supplementary file 1 [file pathogens-10-00131-s001.zip › common_rust_data/train/Early_stage_common_rust/rst (106) - Copy.JPG]

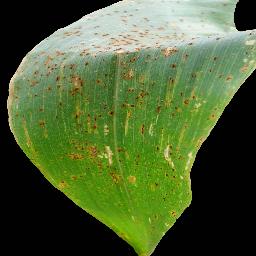

Supplement: Supplementary file 1 [file pathogens-10-00131-s001.zip › common_rust_data/train/Early_stage_common_rust/rst (106).JPG]

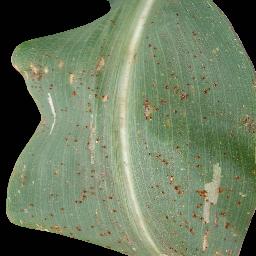

Supplement: Supplementary file 1 [file pathogens-10-00131-s001.zip › common_rust_data/train/Early_stage_common_rust/rst (107) - Copy.JPG]

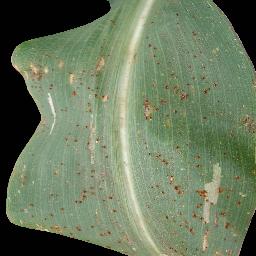

Supplement: Supplementary file 1 [file pathogens-10-00131-s001.zip › common_rust_data/train/Early_stage_common_rust/rst (107).JPG]

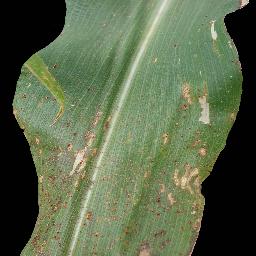

Supplement: Supplementary file 1 [file pathogens-10-00131-s001.zip › common_rust_data/train/Early_stage_common_rust/rst (108) - Copy.JPG]

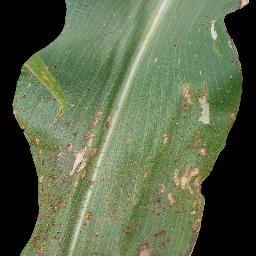

Supplement: Supplementary file 1 [file pathogens-10-00131-s001.zip › common_rust_data/train/Early_stage_common_rust/rst (108).JPG]

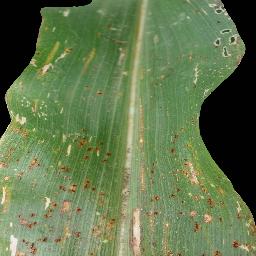

Supplement: Supplementary file 1 [file pathogens-10-00131-s001.zip › common_rust_data/train/Early_stage_common_rust/rst (109) - Copy.JPG]

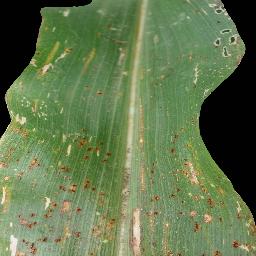

Supplement: Supplementary file 1 [file pathogens-10-00131-s001.zip › common_rust_data/train/Early_stage_common_rust/rst (109).JPG]

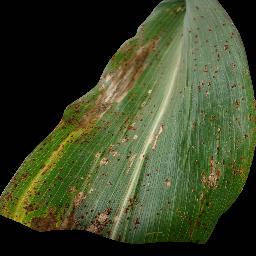

Supplement: Supplementary file 1 [file pathogens-10-00131-s001.zip › common_rust_data/train/Early_stage_common_rust/rst (11) - Copy.JPG]

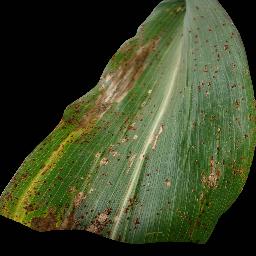

Supplement: Supplementary file 1 [file pathogens-10-00131-s001.zip › common_rust_data/train/Early_stage_common_rust/rst (11).JPG]

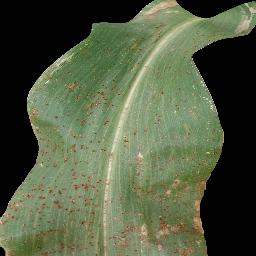

Supplement: Supplementary file 1 [file pathogens-10-00131-s001.zip › common_rust_data/train/Early_stage_common_rust/rst (110) - Copy.JPG]

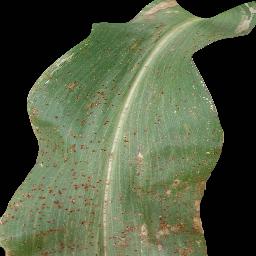

Supplement: Supplementary file 1 [file pathogens-10-00131-s001.zip › common_rust_data/train/Early_stage_common_rust/rst (110).JPG]

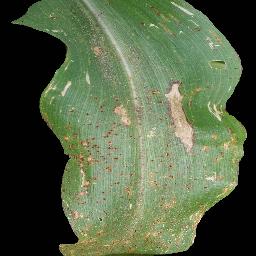

Supplement: Supplementary file 1 [file pathogens-10-00131-s001.zip › common_rust_data/train/Early_stage_common_rust/rst (111) - Copy.JPG]

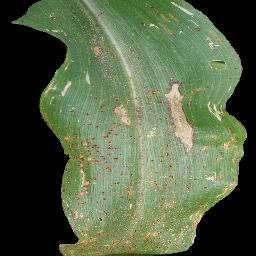

Supplement: Supplementary file 1 [file pathogens-10-00131-s001.zip › common_rust_data/train/Early_stage_common_rust/rst (111).JPG]

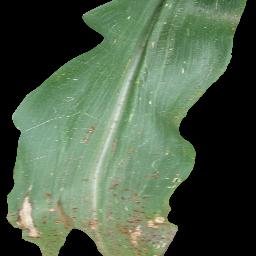

Supplement: Supplementary file 1 [file pathogens-10-00131-s001.zip › common_rust_data/train/Early_stage_common_rust/rst (112) - Copy.JPG]

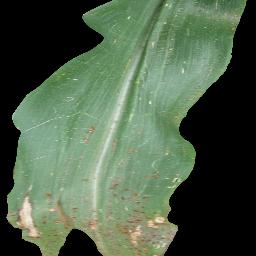

Supplement: Supplementary file 1 [file pathogens-10-00131-s001.zip › common_rust_data/train/Early_stage_common_rust/rst (112).JPG]

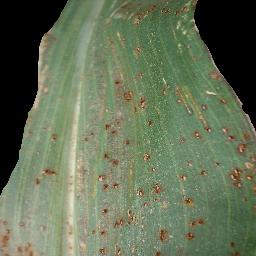

Supplement: Supplementary file 1 [file pathogens-10-00131-s001.zip › common_rust_data/train/Early_stage_common_rust/rst (113) - Copy.JPG]

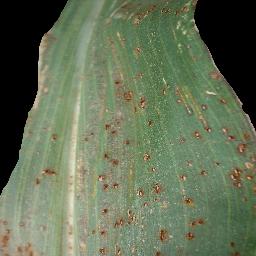

Supplement: Supplementary file 1 [file pathogens-10-00131-s001.zip › common_rust_data/train/Early_stage_common_rust/rst (113).JPG]

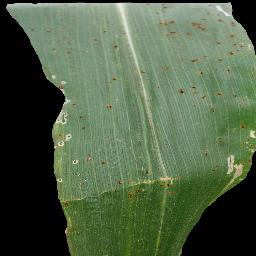

Supplement: Supplementary file 1 [file pathogens-10-00131-s001.zip › common_rust_data/train/Early_stage_common_rust/rst (114) - Copy.JPG]

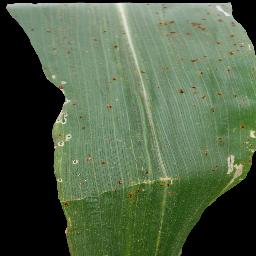

Supplement: Supplementary file 1 [file pathogens-10-00131-s001.zip › common_rust_data/train/Early_stage_common_rust/rst (114).JPG]

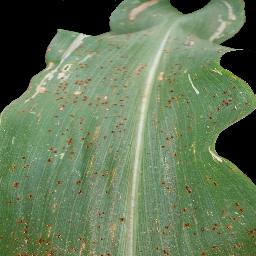

Supplement: Supplementary file 1 [file pathogens-10-00131-s001.zip › common_rust_data/train/Early_stage_common_rust/rst (115) - Copy.JPG]

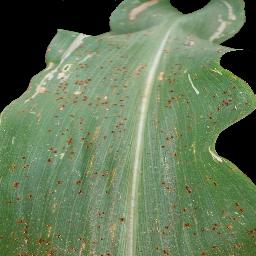

Supplement: Supplementary file 1 [file pathogens-10-00131-s001.zip › common_rust_data/train/Early_stage_common_rust/rst (115).JPG]

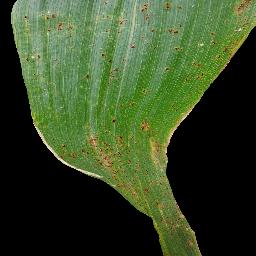

Supplement: Supplementary file 1 [file pathogens-10-00131-s001.zip › common_rust_data/train/Early_stage_common_rust/rst (116) - Copy.JPG]
